# Utilization willingness of institutional care between disabled and non-disabled seniors: evidence from Jiangsu, China

**DOI:** 10.1186/s12913-019-4259-y

**Published:** 2019-06-24

**Authors:** Na Chen, Xin Li, Ni Yuan, Cheng-chao Zhou, Chang-qing Wang

**Affiliations:** 1 School of Health Economics and Management, Nanjing University of Chinese Medicine, Nanjing, 210023 People’s Republic of China; 2 School of Pharmacy, Nanjing Medical University, Nanjing, 211166 People’s Republic of China; 3 School of Public Health, Dalian Medical University, Dalian, 116044 People’s Republic of China; 4 School of Public Health, Shandong University, 44 Wen-hua-xi Road, Jinan, 250100 Shandong China; 5 Nanjing Medical University, 101 Longmian Road, Nanjing, 211166 Jiangsu China

**Keywords:** Willingness, Institutional care, Disabled seniors, Non-disabled seniors, China

## Abstract

**Background:**

Due to rapidly growing number of old adults and diminishing supportive functions of family in China, the issue of willingness to use institutional care is of high priority, especially for disabled seniors. The objective of this study is to compare the willingness of institutional care and its determinants between disabled and non-disabled seniors in China.

**Methods:**

2493 seniors (60+) were randomly selected from a cross-sectional study conducted in three urban districts and three rural counties in Jiangsu Province. Binary logistic regression model was employed to examine differences towards the preference for institutional care between two subgroups, and to identify factors associated with willingness of institutional care between disabled and non-disabled seniors.

**Results:**

Of 2493 respondents, 402 (16.1%) were disabled seniors. Overall, 14.2% of the participants had willingness for institutional care in Jiangsu, China. The willingness for institutional care among non-disabled seniors (OR = 0.513; 95%CI 0.387–9.680) was significantly lower than that among disabled ones. The preference for institutional care of both disabled and non-disabled seniors was associated with household income. The willingness of institutional care was also related to age, education and living arrangement among disabled seniors. Meanwhile, non-disabled seniors who had non-communicable diseases were found to be more likely to choose elder care in institution.

**Conclusions:**

Our findings indicated that the willingness for institutional care among disabled seniors was significantly higher than that among non-disabled ones. Household income was determinant of utilization willingness for institutionalization both in disabled and non-disable seniors. Different policies should be made or modified for disabled and non-disabled seniors separately.

## Background

As the world’s most populous country with the largest number of aging population [1], China has experienced an unprecedented aging process because of extending life expectancy and reducing mortality. In 2016, 16.7% of Chinese population (230.86 million) were aged 60 and above [2]. The older adults are predicted to account for 33% of the total population in China by 2050 [3]. With increasing and aging population, it is a persistent challenge for China to take better care of elders, especially disabled seniors. The disabled seniors refer to those older adults with activity of daily living (ADL) disability, which is assessed by instruments using the International Classification of Functioning, Disability, and Health framework [4]. A nation-wide sample survey indicated that there were 40.63 million disabled seniors in 2015, accounting for 18.3% of the total older adults in China [5].

Despite the increasing percentage of elders, supportive functions of family are diminishing. Filial piety is the basic norm in the Confucian doctrine [6], which means that adult children have responsibility to take care of older people. However, supportive functions of family to take care of older adults are immensely weakened due to decreasing family size and increasing demographic shift [7, 8]. “4:2:1”family structure (four grandparents, two adult children, and one grandchild) poses a dilemma for the adults who have to look after four parents and take care of one child at the same time [9]. The phenomenon is likely to become even more pressing in the near future with the implementation of universal two-child policy.

Due to diminishing supportive functions of family and rapid growth of older adults, especially the increasing percentage of disabled seniors in China [10], exploring the willingness of institutional care and its influential factors are of high priority. Several previous studies have identified determinants of willingness for institutional care, such as age [11, 12], gender [13], income [11], education [12, 14], marital status [11, 12], living arrangements [11–13], non-communicable disease (NCDs) [15], and insurance [16]. However, few studies have explored the willingness of institutional care among disabled seniors [17], and no studies have compared such willingness between disabled and non-disabled seniors in China [18]. To remedy the situation, this study aims to explore the disparity in utilization willingness of institutional care between disabled and non-disabled seniors in China. To do so, we have following specific objectives. First, we will compare the preference for institutionalized care between disabled and non-disabled seniors. Second, we will identify influential factors of the willingness for institutional care among disabled and non-disabled seniors separately.

## Methods

### Study setting and study population

This cross-sectional study was conducted in Jiangsu province. Jiangsu ranks the third in terms of population in China. Moreover, this province has the most percentage of seniors, in which the older adults aged 60 and above account for 21.4% of the total population (about 16.48 million) in 2015 [19]. The percentage is much higher than the national average.

Three-stage cluster sampling method was adopted to select participants. First, we divided the urban districts and rural counties into three categories according to GDP (Gross domestic product) per capita (2015). Then one county and one district were randomly selected from each category. Therefore, three urban counties (Gusu, Qinghe, Haizhou) and three rural districts (Liyang, Xinghua, Xinyi) were confirmed as study sites. Next, three levels of townships and sub-district were randomly stratified from each sampling county and district in the same way. Finally, three villages and three communities were randomly selected from each subgroup. In all, a total of 2700 seniors were recruited, of which 2493 ones with complete data from 27 rural village and 27 urban communities were included in the analysis.

### Data collection

The data was collected from June to August in 2016 by using face-to-face interview. The standard structured questionnaire was composed of social demographic characteristics, living arrangement, health insurance, household income, and willingness of institutional care. In order to ensure quality, the cross-sectional study was conducted by trained postgraduate students from Nanjing University of Chinese Medicine. Participants were informed about the aim of the survey, the selection criterion of the sample, and the assurance that the information was only used for research. Furthermore, the proxy consent procedure was given for those participants who were considered cognitively impaired. All participants gave written informed consent before inclusion in the study. The Cronbach’s Alpha of the total questionnaire is 0.784, indicating a high reliability.

### Variables and measures

#### Dependent variables

The willingness of institutional care was defined as dependent variables, measuring by the question, ‘which way of old-age pension are you willing for?’ If the answer was ‘institutional care’, then ‘yes’ was coded. On the contrary, if the older people choose ‘home-based care’ or ‘community-based care’, the dependent variable was defined as ‘no’.

#### Independent variables

On the basis of Andersen-Nyman model [18, 20, 21], we classified explanatory factors into three categories and choosing appropriate variables: 1) *Predisposing variables*, including gender (female, male), age (60–69, 70–79,80+), marital status (married or others), and education (primary school or below, junior school, high school or above). 2) *Enabling variables,* including residence (urban or rural), living arrangements (living alone, living with children or others), health insurance (New cooperative medical scheme (NCMS), Medical insurance for urban residents’ scheme (MIUR), Medical insurance for urban employee scheme (MIUE), others including commercial insurance), and household income (Q1, Q2, Q3 andQ4). According to the Jiangsu Statistical Yearbook, we adopted quartile to divide the household income into four groups. Quartile 4 (Q4) is the richest and Quartile 1 (Q1) is the poorest. 3) *Need variables*, consisted of ADL disability (yes or no) and NCDs including hypertension, diabetes, Heart disease, stroke, rheumatism, Alzheimer’s disease et al. (yes or no).

The ADL disability was measured by Katz Index Scale [22–25], which included the following six items: feeding, dressing, bathing, toileting, walking inside, bladder and bowel control. Each item had two response choices: “dependent” and “independent”. If any answer was “dependent”, the seniors were categorized as older adults with ADL disability which means needing equipment or human help to complete the above task, otherwise, the seniors were defined as non-disabled ones without ADL disability. The Chinese version scale was proven to be of good validity and reliability [26].

### Statistical analysis

The data were double entered and checked by Epi Data V.3.02. The statistical package SPSS V.20.0 was employed to analyze the data. Chi-square test was performed to examine differences in the independent variables mentioned above between disabled and non-disabled seniors. Binary logistic regression was applied to assess the association of willingness to live in institution with the ADL disability among old adults, and to identify the explanatory factors between disabled and non-disabled seniors separately. All reported CIs were calculated at the 95% level. Statistical significance was set at the 5% level.

## Results

### Basic information of the participants

As shown in Table 1, 14.2% of the participants had willingness for institutional care in Jiangsu, China. The proportion of disabled and non-disabled seniors who preferred institutional care was 22.6 and 12.6% separately. Of all respondents, the majority of seniors were non-disabled (83.9%), at the ages of 70–79 (51.3%), female (52.7), married (75.2%), having education level of primary or below (64.4%), rural (63.5%), living with children or others (86.8%), covered by NCMS (55.7%), the poorest (47.9%), and having NCDs (87.3%). In general, age (*P* < 0.001), marital status (*P* < 0.001), living arrangement (*P* < 0.001), household income (*P* = 0.002) and NCDs (*P* < 0.001) were significantly different between non-disabled and disabled seniors.

**Table 1:** Characteristics of the sample in Jiangsu, China (2016)

Characteristics	Total		Non-disabled seniors		Disabled seniors		*P*
	n	%	n	%	n	%	
Observation	2493	100.0	2091	83.9	402	16.1	
Willingness of institutional care	355	14.2	264	12.6	91	22.6	< 0.001
Predisposing factors							
Age							< 0.001
60–69	858	34.4	773	37.0	85	21.1	
70–79	1278	51.3	1083	51.8	195	48.5	
80+	357	14.3	235	11.2	122	30.4	
Gender							0.413
Female	1314	52.7	1110	53.1	204	50.7	
Male	1179	47.3	981	46.9	198	49.3	
Marital status							< 0.001
Married	1874	75.2	1619	77.4	255	63.4	
Others	619	24.8	472	22.6	147	36.6	
Education							0.091
Primary or below	1606	64.4	1328	63.5	278	69.2	
Junior	581	23.3	498	23.8	83	20.6	
High or above	306	12.3	265	12.7	41	10.2	
Enabling factors							
Residence							0.101
Urban	909	36.5	777	37.2	132	32.8	
Rural	1584	63.5	1314	62.8	270	67.2	
Living arrangements							< 0.001
Alone	330	13.2	243	11.6	87	21.6	
With children or others	2163	86.8	1848	88.4	315	78.4	
Insurance ^a^							0.335
None	96	3.9	74	3.5	22	5.5	
MIUE	340	13.6	291	13.9	49	12.2	
MIUR	595	23.9	499	23.9	96	23.9	
NCMS	1389	55.7	1168	55.9	221	55.0	
Others	73	2.9	59	2.8	14	3.5	
Household income ^b^							0.002
Q_4_	117	4.7	100	4.8	17	4.2	
Q_3_	327	13.1	268	12.8	59	14.7	
Q_2_	855	34.3	749	35.8	106	26.4	
Q_1_	1194	47.9	974	46.6	220	54.7	
Need factors							
NCD ^c^							< 0.001
Yes	2176	87.3	1797	85.9	379	94.3	
No	317	12.7	294	14.1	23	5.7	

^a^*NCMS* New cooperative medical scheme, *MIUR* Medical insurance for urban residents scheme, *MIUE* Medical insurance for urban employee scheme

^b^Quartile 4 (Q4) is the richest and Quartile 1 (Q1) is the poorest

^c^*NCD* Non-communicable chronic disease

### Association of willingness for institutional care and ADL disability among seniors

Table 2 showed the disparity of willingness to live in institution between disabled and non-disabled seniors in two models. Results from model 1 identified that willingness was statistically higher among disabled seniors (OR = 0.494, 95CI 0.378–0.645, *P* < 0.001). Model 2 indicated that when controlling other variables, willingness of institutional care among disabled seniors was also significantly higher than that among non-disabled seniors (OR = 0.513, 95CI 0.387–9.680, *P* < 0.001).

**Table 2:** Association of ADL disability and willingness for institutional care among seniors in Jiangsu, China,2016

Characteristics	Model 1(No covariates)		Model 2 (Covariates)	
	OR (95% CI)	*P*	OR (95% CI)	*P*
ADL disability				
No	0.494(0.378–0.645)	< 0.001	0.513(0.387–9.680)	< 0.001
Yes	1.0		1.0	
Age				
60–69			2.003(1.339–2.997)	0.001
70–79			1.650(1.130–2.410)	0.010
80+			1.0	
Gender				
Male			1.122(0.880–1.430)	0.352
Female			1.0	
Marital status				
Married			0.806(0.598–1.087)	0.158
Others			1.0	
Education				
Primary or below			0.615(0.412–0.918)	0.017
Junior			0.714(0.476–1.070)	0.103
High or above			1.0	
Residence				
Urban			1.053(0.771–1.437)	0.747
Rural			1.0	
Living arrangements				
Alone			1.542(1.096–2.170)	0.013
With children or others			1.0	
Insurance ^a^				
None			0.679(0.320–1.442)	0.314
MIUE			0.883(0.437–1.783)	0.729
MIUR			0.940(0.477–1.852)	0.859
NCMS			1.158(0.498–2.694)	0.734
Others			1.0	
Household income ^b^				
Q_4_			0.918(0.508–1.657)	0.776
Q_3_			1.008(0.682–1.490)	0.969
Q_2_			0.616(0.458–0.828)	0.001
Q_1_			1.0	
NCD ^c^				
No			0.420(0.266–0.663)	< 0.001
Yes			1.0	

^a^*ADL* activity of daily living

^b^*NCMS* New cooperative medical scheme, *MIUR* Medical insurance for urban residents scheme, *MIUE* Medical insurance for urban employee scheme

^c^Quartile 4 (Q4) is the richest and Quartile 1 (Q1) is the poorest

^d^*NCD* Non-communicable chronic disease

### Factors associated with willingness for institutional care among disabled seniors

We identified the factors associated with the willingness to live in institution among disabled seniors (Table 3). Univariate analysis showed that eight factors were found to be statistically significant (*P* < 0.05) related to the willingness of institutional care, including age, gender, marital status, education status, residence, household income. Multi-logistic regression showed that disabled seniors who were aged from 60 to 80 (*P* < 0.001), who lived alone (*P* = 0.023), whose household income was corresponding to Q3 (*P* = 0.025) preferred choosing institutional care, and those who had lower educational level (*P* < 0.001) did not prefer institutional care.

**Table 3:** Factors associated with willingness of institutional care among disabled seniors in Jiangsu, China, 2016 (*n* = 402)

Characteristics	Willingness of institutional care		OR _C_ (95%CI)	*P*	OR _a_ (95%CI)	*P*
	No (%)	Yes (%)				
*N* = 402	311(77.4)	91(22.6)				
Age						
60–69	61(71.8)	24(28.2)	3.299(1.567–6.943)	0.002	4.310(1.882–9.869)	0.001
70–79	141(72.3)	54(27.2)	3.211(1.668–6.182)	< 0.001	3.836(1.842–7.985)	< 0.001
80+	109(89.3)	13(10.7)	1		1	
Gender						
Male	143(72.2)	55(27.8)	1.795(1.115–2.888)	0.016	1.629(0.956–2.774)	0.072
Female	168(82.4)	36(17.6)	1		1	
Marital status						
Married	194(76.1)	61(23.9)	1.795(1.115–2.888)	0.016	1.213(0.633–2.326)	0.561
Others	117(79.6)	30(20.4)	1		1	
Education						
Primary or below	225(80.9)	53(19.1)	0.151(0.075–0.302)	< 0.001	0.200(0.083–0.480)	< 0.001
Junior	70(84.3)	13(15.7)	0.119(0.050–0.282)	< 0.001	0.107(0.041–0.276)	< 0.001
High or above	16(39.0)	25(61.0)	1		1	
Residence						
Urban	93(70.5)	39(29.5)	1.758(1.087–2.844)	0.021	1.169(0.653–2.096)	0.599
Rural	218(80.7)	52(19.3)	1		1	
Living arrangements						
Alone	61(70.1)	26(29.9)	1.639(0.961–2.796)	0.070	2.218(1.118–4.402)	0.023
With children or others	250(79.4)	65(20.6)	1		1	
Insurance ^a^					NA	
MIUR	65(67.7)	31(32.3)	1.648(0.743–3.651)	0.219		
NCMS	178(80.5)	43(19.5)	0.835(0.395–1.765)	0.636		
None	18(81.8)	4(18.2)	0.768(0.215–2.746)	0.684		
Others	12(85.7)	2(14.3)	0.576(0.112–2.970)	0.510		
MIUE	38(77.6)	11(22.4)	1			
Household income ^b^						
Q_4_	11(64.7)	6(35.3)	2.312(0.809–6.606)	0.118	1.062(0.271–4.154)	0.932
Q_3_	35(59.3)	24(40.7)	2.906(1.565–5.396)	0.001	2.512(1.126–5.607)	0.025
Q_2_	87(82.1)	19(17.9)	0.926(0.508–1.686)	0.800	0.697(0.355–1.367)	0.294
Q_1_	178(80.9)	42(19.1)	1		1	
NCD ^c^					NA	
No	21(91.3)	2(8.7)	0.310(0.071–1.349)	0.119		
Yes	290(76.5)	89(23.5)	1			

*OR*_*C*_ crude odds ratio

*OR*_*a*_ adjusted odds ratio

^a^*NCMS* New cooperative medical scheme, *MIUR* Medical insurance for urban residents scheme, *MIUE* Medical insurance for urban employee scheme

^b^Quartile 4 (Q4) is the richest and Quartile 1 (Q1) is the poorest

^c^*NCD* Non-communicable chronic disease

### Factors associated with willingness for institutional care among non-disabled seniors

The influential factors associated with willingness of institutional care among non-disabled seniors showed in Table 4. The univariate analysis indicated that the non-disabled seniors who were married (*P* = 0.006), whose household income belonged to Q2 (*P* = 0.001), who had no NCDs (*P* = 0.002) were less likely to choose institutional care. Those who lived alone (*P* = 0.007), who were not covered by any medical insurance (*P* = 0.022) preferred institutional care. Multi-logistic regression showed that two factors from the above five items were still significantly associated with the willingness, including NCDs and household income.

**Table 4:** Factors associated with willingness of institutional care among non-disabled seniors in Jiangsu, China, 2016 (*n* = 2091)

Characteristics	Willingness of institutional care		OR _C_ (95%CI)	*P*	OR _a_ (95%CI)	*P*
	No (%)	Yes (%)				
*N* = 2091	1827(87.4)	264(12.6)				
Age					NA	
60–69	666(86.2)	107(13.8)	1.188(0.762–1.852)	0.448		
70–79	954(88.1)	129(11.9)	1.000(0.647–1.545)	0.999		
80+	207(88.1)	28(11.9)	1			
Gender					NA	
Male	863(88)	118(12)	0.903(0.697–1.170)	0.440		
Female	964(86.8)	146(13.2)	1			
Marital status						
Married	1432(88.4)	187(11.6)	0.670(0.502–0.893)	0.006	0.770(0.553–1.072)	0.121
Others	395(83.7)	77(16.3)	1		1	
Education					NA	
Primary or below	1159(87.3)	169(12.7)	1.234(0.808–1.885)	0.330		
Junior	431(86.5)	67(13.5)	1.316(0.823–2.102)	0.251		
High or above	237(89.7)	28(10.6)	1			
Residence					NA	
Urban	689(88.7)	88(11.3)	0.826(0.629–1.085)	0.169		
Rural	1138(86.6)	176(13.4)	1			
Living arrangements						
Alone	199(81.9)	44(18.1)	1.636(1.147–2.335)	0.007	1.392(0.927–2.088)	0.111
With children or others	1628(88.1)	220(11.9)	1		1	
Insurance ^a^						
MIUR	443(88.8)	56(11.2)	1.236(0.762–2.005)	0.391	1.145(0.698–1.877)	0.592
NCMS	1010(86.5)	158(13.5)	1.530(0.995–2.351)	0.053	1.274(0.792–2.048)	0.317
Others	50(84.7)	9(15.3)	1.760(0.781–3.967)	0.173	1.643(0.719–3.752)	0.239
None	60(81.8)	14(18.9)	2.281(1.129–4.612)	0.022	1.896(0.906–3.965)	0.089
MIUE	264(90.7)	27(9.3)	1			
Household income ^b^						
Q_4_	88(88)	12(12)	0.761(0.406–1.426)	0.394	0.887(0.459–1.715)	0.722
Q_3_	237(88.4)	31(11.6)	0.730(0.483–1.104)	0.136	0.851(0.542–1.336)	0.483
Q_2_	676(90.3)	73(9.7)	0.603(0.447–0.812)	0.001	0.657(0.477–0.905)	0.010
Q_1_	826(84.8)	148(15.2)	1		1	
NCD ^c^						
No	274(93.2)	10(6.8)	0.465(0.289–0.746)	0.002	0.450(0.279–0.725)	0.001
Yes	1553(86.4)	244(13.6)	1		1	

*OR*_*C*_ crude odds ratio

*OR*_*a*_ adjusted odds ratio

^a^*NCMS* New cooperative medical scheme, *MIUR* Medical insurance for urban residents scheme, *MIUE* Medical insurance for urban employee scheme

^b^Quartile 4 (Q4) is the richest and Quartile 1 (Q1) is the poorest

^c^*NCD* Non-communicable chronic disease

## Discussion

Based on the cross-sectional study conducted in Jiangsu,, our study compared the willingness of institutional care between disabled and non-disabled seniors in China and explored determinants in two subgroups separately. To our knowledge, it is the first study that focuses on the research subject above. Our findings showed that the willingness for institutional care among disabled seniors was significantly higher than non-disabled old adults.

Our study found that 14.2% of seniors chose institutional care. The percentage was lower than 45% which was reported in America [27] and 16.7% demonstrated in Taiwan [28]. Compared with studies of other provinces, the proportion was also lower than Beijing (30%) [29], Henan (16.1%) [16] and Guizhou (38.5%) [14]. However, it was higher than Shandong (8.5%) [30] and Zhejiang province [31]. In different circumstance, there is a large disparity of preference for institutional care among the seniors.

Consistent with other studies [32, 33], the result of our study demonstrated there was a significant difference in willingness of institutional care between two subgroups. The proportion of disabled and non-disabled seniors who preferred institutional care was 22.6 and 12.6% separately. However, another study [18] which revealed that disabled seniors were less likely to choose institutional care. One possible explanation is that attitudinal changes towards institutional care is happening in China in recent years [34]. More and more aging adults, especially disabled seniors recognize that institutional care is a good option for them because of the poor physical status, diminishing family size and reducing care functions [17, 32]. Another explanation may be the improvement of institutional service for seniors. Particularly, integrated care in some nursing home which combined medical care with nursing services is preferred by disabled seniors with high household income. Furthermore, the support of government is a favorable objective factor. This finding suggests that it is urgent to make and modify different supporting policy for disabled and non-disabled seniors separately.

Similarly, the finding in this study was that household income level significantly determined the utilization willingness of institutional care both in disabled older adults whose household income was corresponding to Q3 and non-disabled seniors whose household income belonged to Q2. There are two possible explanations for such output. First, older adults with high household income would pay more attention to health status and quality of life, so the institution with better medical care and nursing service [35, 36] is favored by older adults, especially the disabled ones with poor mental and physical condition. However, disabled seniors with higher household income which are enough to afford private professional care may have other options other than institutional care [37]. Second, many poor seniors have to give up their idea of institutional care because of financial insolvency, but if older people are the poorest ones, the willingness of institutional care may increase because the country is guaranteeing the rights of them through policy orientation. The finding indicates that maybe the government should pay special attention to the willingness of institutional care among middle-income seniors, which is an important question worthy of further exploration.

Furthermore, the study demonstrated that the preference for institutional care of disabled seniors was also found to be significantly related to three indicators including age, living arrangement, and education, which was consistent with previous studies [17, 18]. Education exerts a statistically positive effect on the willingness. The seniors with better education are open-minded, who will be easily to accept the new idea in view of diminishing family size and increasing geographic mobility [35]. In addition, disabled seniors living alone were more likely to choose institutional care because they find it hard to obtain informal support [30, 38].

Interestingly, age had a negative impact on the willingness for institutional care among disabled seniors. Essentially, with the growth of age, physical and psychological health of old adults become poor, leading to high demand and willingness of institutional care. However, the result of the study is opposite, which may be explained by Chinese traditional culture and changes of family structure [39]. Most of the oldest old tend to reject institutional care because of traditional family norms, intense loneliness, and extreme insecurity causes. Meanwhile, the young older people are more likely to accept the concept of modern pension institution [40]. The findings should, therefore, give an impetus to attach importance to the traditional culture and family concept in the study of willingness to live in eldercare institution.

Parallel to other studies [15, 18], we found that the non-disabled seniors who suffered from NCDs were more likely to prefer institutional care. The possible reason is that some NCDs are strongly related to worse physical condition which needs professional institutional service, including dementia, cardiovascular diseases and arthritis [41, 42]. According to the WHO Global Status Report (2010), NCDs are the leading causes of mortality and disability globally [43]. China, as the most populous developing country, has experienced the heavy burden of NCDs, accounting for 70% of total disease burden and 80% of total death [44]. However, for disabled seniors, 94.3% among them had NCDs according to the sample, leading to the possibility of neglecting the effect of NCDs. The finding shows that it is urgent to do further research to have a better understanding of epidemiology of NCDs among older adults.

This study has some limitations. Firstly, the information including the economic status and willingness of institutional care were self-reported, leading to the possibility of bias. Secondly, a cross-sectional design was adopted in our study to illustrate the relationship between willingness of institutional care and ADL disability difference rather than causality. Thirdly, some important variables are unavailable in the current study, such as the understanding of the institutional care [45], information of the offspring [17] and the psychological status [13, 16], which are the research directions in the future..

## Conclusion

This study showed that the willingness for institutional care among disabled seniors was significantly higher than that among non-disabled ones. Household income was found to be an important determinant for the willingness in both disabled and non-disable seniors. Additionally, age, living arrangement, and education were predictors for willingness among disabled seniors. Non-disabled seniors who had NCDs were more likely to choose institutional care. According to the findings, several countermeasures should be put forward. Targeting policies should be made or modified to satisfy various demands of the disabled and non-disabled seniors separately.

## Data Availability

The datasets used and/or analyzed during the current study are available from the corresponding author on reasonable request.

## Abbreviations

ADL: Activity of Daily Living
MIUE: Medical Insurance for Urban Employee Scheme
MIUR: Medical Insurance for Urban Residents Scheme
NCDs: Non-Communicable Disease
NCMS: New Cooperative Medical Scheme
OR_a_: Adjusted Odds Ratio
OR_C_: Crude Odds Ratio
